# High truncated-O-glycan score predicts adverse clinical outcome in patients with localized clear-cell renal cell carcinoma after surgery

**DOI:** 10.18632/oncotarget.15900

**Published:** 2017-03-04

**Authors:** SonTung NguyenHoang, Yidong Liu, Le Xu, Lin Zhou, Yuan Chang, Qiang Fu, Zheng Liu, Zongming Lin, Jiejie Xu

**Affiliations:** ^1^ Department of Urology, Zhongshan Hospital, Fudan University, Shanghai, China; ^2^ Department of Biochemistry and Molecular Biology, School of Basic Medical Sciences, Fudan University, Shanghai, China; ^3^ Department of Urology, Ruijin Hospital, School of Medicine, Shanghai Jiaotong University, Shanghai, China

**Keywords:** localized clear-cell renal cell carcinoma, truncated O-glycans, overall survival, recurrence-free survival, prognosticator

## Abstract

Truncated O-glycans, including Tn-antigen, sTn-antigen, T-antigen, sT-antigen, are incomplete glycosylated structures and their expression occur frequently in tumor tissue. The study aims to evaluate the abundance of each truncated O-glycans and its clinical significance in postoperative patients with localized clear-cell renal cell carcinoma (ccRCC). We used immunohistochemical testing to analyze the expression of truncated O-glycans in tumor specimens from 401 patients with localized ccRCC. Truncated-O-glycan score was built by integrating the expression level of Tn-, sTn- and sT-antigen. Kaplan-Meier survival and Cox regression analysis were done to compare clinical outcomes in subgroups. Receiver operating characteristic (ROC) was applied to assess the impact of prognostic factors on overall survival (OS) and recurrence-free survival (RFS). The results identified Tn-, sTn-, sT-antigen as independent prognosticators. The OS and RFS were shortened among the 198 (49.4%) patients with high Truncated-O-glycan score than among the 203 (50.6%) patients with low score (hazard ratio for OS, 7.060; 95% confidence interval [CI]: 2.765 to 18.027; *p* <0.001; for RFS, 4.612; 95% CI: 2.141 to 9.931; *p* <0.001). There is no difference between low-risk patients and high-risk patients in low score group (*p* = 0.987). High-risk patients with low score showed a better prognosis than low-risk patient with high score (*p* = 0.029). The Truncated-O-glycan score showed better prognostic value for OS (AUC: 0.739, *p* = 0.003) and RFS (AUC: 0.719, *p* = 0.003) than TNM stage. In summary, the high Truncated-O-glycan score could predict adverse clinical outcome in localized ccRCC patients after surgery.

## INTRODUCTION

Renal cell carcinoma (RCC), which accounts for 2%-3% of all adult malignant neoplasm, is one of the most common urologic cancer [1]. Clear cell RCC (ccRCC), formally known as “conventional” RCC, accounts for 70%-80% of all RCC. Traditionally, 30%-40% of patients with RCC died of cancer, in contrast to the 20% mortality rate associated with prostate cancer and bladder carcinoma [2]. In general, patients with ccRCC have worse clinical outcomes compared with papillary or chromphobe RCC which are the other two most common histologic subtype of RCC. Outcomes for patients with similar clinical and pathological features still differ significantly. Currently, TNM stage, Fuhrman grade, and Eastern Cooperative Oncology Group performance status (ECOG-PS) remain the most commonly used predictors of survival for patients with ccRCC. Even though several integrated prognostic models have been established to predict clinical outcomes [3, 4], such as University of California Integrated Staging System (UISS) [5], Mayo Clinic Stage Size Grade and Necrosis (SSIGN) score [6] and Leibovich score [3], prognosis after nephrectomy is not satisfactory since high incidence of local recurrence or distant metastasis and a low 5-year survival rate.

Changes in cellular glycosylation profiles are deeply involved in embryonic development, cellular activation and intercellular interaction in vertebrates and therefor it is not surprising that glycosylation changes are universal features during malignant transformation and tumor progression [7-9]. ccRCC are currently recognized as a metabolic disease [10] which possibly associated with some aberrant modifications of carbohydrate antigen expression. Such carbohydrate epitopes could be favorable markers for identifying distinct subtypes of disease as well as a potential approach to well predict clinical outcomes.

In last few decades, a large amount of studies had been paying attention to glycosylation modification in tumor. The glycosylation could be changed in various ways during malignant transformation and development. One of most common consequence is the expression of truncated O-glycans which occur infrequently in normal cells and tissues. Truncated O-glycans mainly consist of 4 carbohydrate epitopes, including: Tn-antigen, sTn-antigen, T-antigen, sT-antigen. The discovery of Tn-antigen associated with human disorders, Tn syndrome [11, 12], was first described in 1957 by Moreau et al. After glycoprotein was produced in endoplasmic reticulum (ER) and transmitted to Golgi apparatus, the biosynthesis of the Tn-antigen is initiated through transferring the GalNAc unit to serine/threonine by polypeptide polypeptide a-N-acetylgalactosaminyltransferases (ppGalNAcTs). Following its formation in Golgi apparatus, the Tn-antigen can severe as a precursor for at least three structures: *(1) T antigen*: T-synthase transfers Gal from donor to Tn antigen to form T-antigen, (2) *Sialyl-Tn antigen (sTn-antigen)*, which is synthesized by ST6GalNac-I, and *(3) core 3 structure* which is a minority group in O-glycan. Beside the sTn-antigen, Sialyl-T antigen (sT-antigen) is another sialylation structure mainly formed by ST6GalNac-II. Among previous studies, the change of Truncated-O-glycan expression that are observed in tumor cells make the glycoproteins and glycolipids expressed by these cells obviously distinct from those expressed by the normal tissues [7]. As a result, we paid attention to Tn-, sTn-, T- and sT-antigen. Cumulative studies showed that 80% of the tumors of the colon, lung, breast, cervix, ovary and prostate express Tn-antigen [7, 13, 14]. However, none of these studies assessed the association of truncated O-glycans expression with clinical outcomes. The study aims to evaluate the abundance of each truncated O-glycans and clinical significance in patients with localized clear-cell renal cell carcinoma (ccRCC).

To address such issues, we adopted immunohistochemical testing to analyze the expression of truncated O-glycans and its association with clinicopathological characteristics and clinical outcomes, including OS and RFS. These results lead us to combine these independent prognosticators for constructing a weighted formula, named Truncated-O-glycan score. Furthermore, we assessed the prognostic value of Truncated-O-glycan score in over cohort and subgroups. Finally, we compared the predictive sensitivity and specificity of Truncated-O-glycan score with TNM stage.

## RESULTS

### Immunohistochemical findings

**Figure 1:**
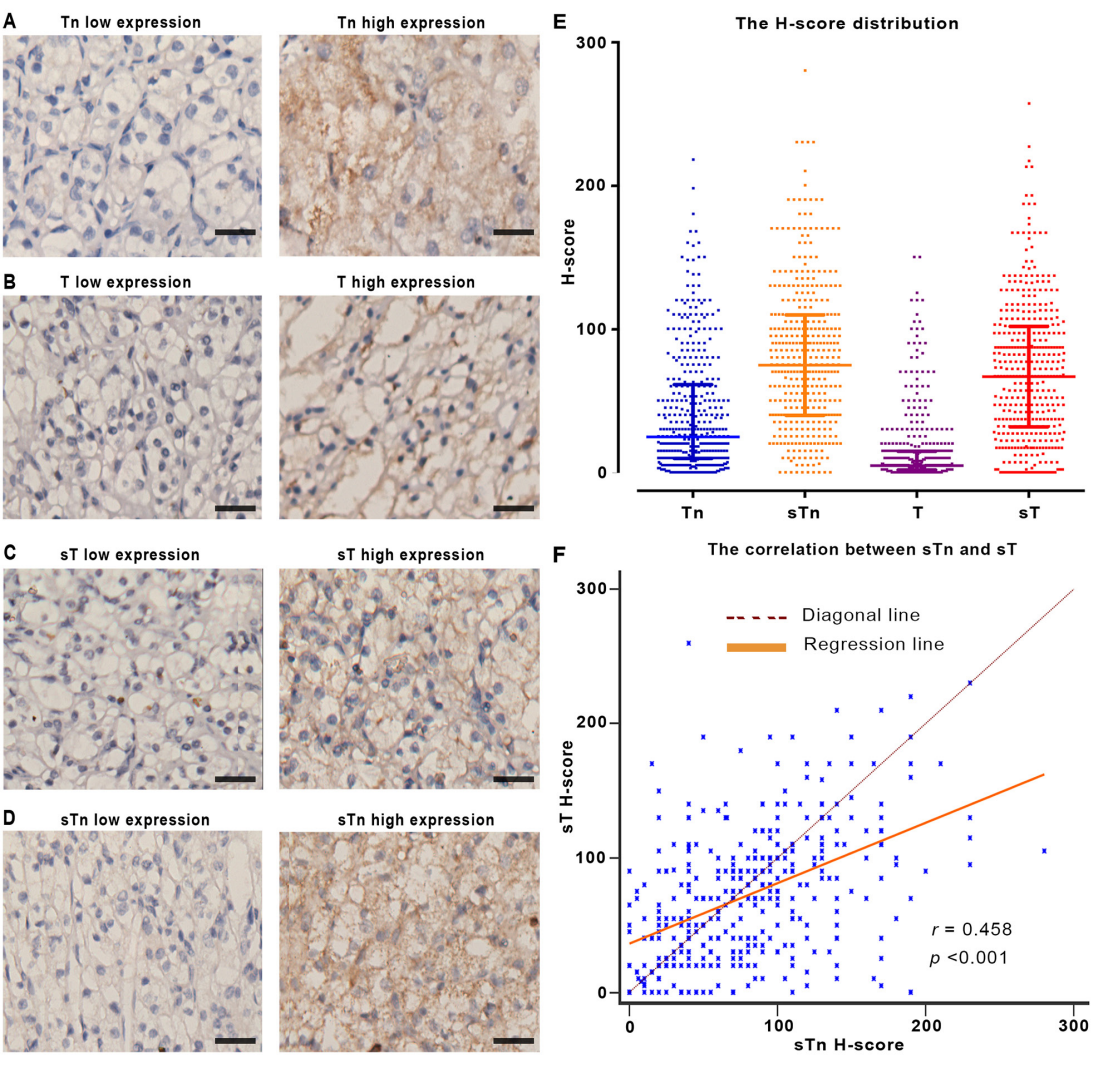
The expression of truncated O-glycans in FFPE tissues **A.**-**D.** Representative IHC image of truncated O-glycans expression. **E.** The column scatterplot shows the H-score distribution of truncated O-glycans. **F.** The scatter diagram shows the relation between sTn and sT expression. Note: Scale bar: 40μm (original magnification × 200). In panel E and F, each square in the column scatterplot represents one patient sample, *r* as regression coefficient, *p* < 0.05 is considered statistically significant.

We firstly evaluated the expression of truncated O-glycans by IHC analysis in the clinical specimens of 401 patients. The specific expression of truncated O-glycans was observed in both cell membrane and intracellular space (Figure 1A, 1B, 1C, 1D). The expression of truncated O-glycans in peritumorous area was much lower than in tumor tissues (Tn antigen: *p* < 0.001; sTn: *p* < 0.001; T-antigen: *p* < 0.001; sT-antigen: *p* < 0.001; Supplementary Figure S1). The relevant H-score have been also provided in order to clarify the difference between high and low levels of expression (Supplementary Figure S2). The difference between H-score of Tn-, T-, sTn- and sT-antigen was showed in Figure 1E. As a result, the expression of T-antigen (H-score median = 5.00) is much weaker than other epitopes’ expression. Additionally, there is a strong correlation between sTn-antigen and sT-antigen (H-score median sTn = 75.00, sT = 67.00, r = 0.458, *p* < 0.001; Figure 1F).

### Truncated O-glycans expression and clinicopathological feature associations

The association between Tn-, T-, sTn- and sT-antigen expression and clinicopathological features was fully summarized (Table 1). Patients with high Tn-antigen expression tended to have high Fuhrman grade, necrosis rate and sacromatoid risk (*p* = < 0.001, 0.001 and 0.036, respectively; Table 1). High sTn-antigen expression tumors tended to have a greater tumor size, higher Fuhrman grade, higher necrosis rate and higher ECOG-PS (*p =* 0.027, 0.001, 0.035 and < 0.001, respectively; Table 1). There is no significant correlation between T-antigen with any clinicopathological features. Patients with low sT-antigen expression tended to have smaller tumor size, lower necrosis rate and lower TNM stage (*p* = 0.032, 0.013 and 0.017, respectively; Table 1).

**Table 1:** Correlation between clinicopathological characteristics and truncated O-glycans expression.

Characteristics	Tn expression		P^*^	sTn expression		P^*^	T expression		P^*^	sT expression		P^*^
	Low(n= 201)	High (n= 200)		Low(n= 204)	High (n= 197)		Low(n=222 )	High (n= 179)		Low(n= 205)	High (n= 196)	
Age at surgery, year Mean ± SD^¶^	54.94 ± 12.92	57.09 ± 10.75	**0.013**	54.77 ± 11.390	57.29 ± 12.35	0.146	55.55 ± 11.85	56.59 ± 12.02	0.771	55.98 ± 12.14	56.05 ± 11.72	0.662
Gender Female Male	68 133	51 149	0.068	62 142	57 140	0.749	70 152	49 130	0.365	72 133	47 149	**0.015**
Tumor size, cm Mean ± SD^¶^	3.95 ± 2.12	4.67 ± 2.57	0.067	3.92 ± 2.07	4.70 ± 2.61	**0.027**	4.48 ± 2.46	4.09 ± 2.26	0.127	4.01 ± 2.16	4.61 ± 2.55	0.254
pT-stage T1+T2 T3+T4	155 46	149 51	0.541	158 46	146 51	0.435	164 58	140 39	0.313	157 48	147 49	0.711
TNM stage Stage I + Stage II Stage III + Stage IV	155 46	151 49	0.704	158 46	148 49	0.584	165 57	141 38	0.298	158 47	148 48	0.713
Fuhrman grade Grade 1 + grade 2 Grade 2 + grade 3	153 48	107 93	**<0.001**	148 56	112 85	**0.001**	148 74	112 67	0.393	138 67	122 74	0.288
LVI^§^ Absent Present	161 40	144 56	0.053	158 46	147 50	0.524	168 54	137 42	0.865	164 41	140 55	0.055
Necrosis Absent Present	177 24	151 49	**0.001**	175 29	153 44	**0.035**	184 38	144 35	0.530	172 33	156 40	0.263
Sarcomatoid Absent Present	199 2	192 8	**0.046**	201 3	190 7	0.181	219 3	172 7	0.101	201 4	190 6	0.476
ECOG-PS^‡^ 0 ≥1	174 27	165 35	0.259	186 18	153 44	**<0.001**	187 35	152 27	0.851	173 32	166 30	0.933
Surgical type Partial Nephrectomy Radical nephrectomy Laparoscopic radical nephrectomy	103 93 5	81 113 81	0.097	94 104 6	90 102 5	0.963	105 112 5	79 94 6	0.692	84 115 6	100 91 5	0.130

*P**-**value <0.05 was regarded as statistically significant;

^¶^ The results of continuous variables are presented as mean ± SD (standard deviation);

^§^LVI = lymphovascular invasion; ^‡^ ECOG-PS = Eastern Cooperative Oncology Group performance status.

### Tn-, sTn- and sT-antigen as independent prognosticators of poor prognosis

Kaplan-Meier curves and log-rank test were done to evaluate the correlation between truncated O-glycans expression and clinical outcomes in localized ccRCC. The median follow-up was 73 months (rang: 39-74 months). As shown in Supplementary Figure S3, high Tn-, sTn- or sT-antigen expression tumors were associated with a worse prognosis than were low expression tumors (OS, *p* < 0.001; RFS, *p* < 0.001). However, there is no significant relation between T-antigen and patient outcomes (OS, *p* = 0.308; RFS, *p* = 0.746). Thus, the expression of Tn-, sTn- or sT-antigen is possible to be considered as significant and powerful prognostic factors.

T-antigen was excluded from Cox regression analysis since lacking of influence on clinical outcomes. Univariate Cox regression analysis was applied to evaluate the prognostic significance of clinicopathological features in localized ccRCC (data not show). The multivariate Cox regression analysis was completed when gender and age at surgery were excluded. In the analysis of overall survival, the hazard ratio for death of Tn-, sTn- and sT-antigen was 4.016 (95% CI: 1.760 to 9.166; *p* = 0.001), 3.017 (95% CI: 1.514 to 6.013; *p* = 0.002), 2.263 (95% CI: 1.208 to 4.239; *p* = 0.011), respectively; and in the analysis of recurrence-free survival, the hazard ratio for recurrence was 2.019 (95% CI: 1.049 to 3.885; *p* = 0.035), 3.292 (95% CI: 1.697-6.385; *p* < 0.001), 2.031 (95% CI: 1.137 to 3.629; *p* = 0.07; Supplementary Table S1). The identical results have been observed when enrolled Tn-, sTn- and sT-antigen simultaneously (Supplementary Table S2). Overall, such results indicate that Tn-, sTn- and sT-antigen could be independent prognosticators of poor prognosis.

### Truncated-O-glycan score construction and its association with localized ccRCC

The construction of Truncated-O-glycan score was fully described in Methods section. The association of Truncate-O-glycan with clinicopathological features was fully displayed (Supplementary Table S3). As a result, high Truncated-O-glycan score tumors is more common among tumor with high Fuhrman grade (*p <* 0.001) and high necrosis risk (*p =* 0.01).

Following Truncated-O-glycan score construction, we applied Harrell C-index to further evaluate the prognostic power of Truncated-O-glycan score compared to Tn-, sTn- and sT-antigen. As displayed in Supplementary Table S4, the prognostic power of Truncate-O-glycan score for OS and RFS is much stronger than any independent antigen expression even though continuous or discontinuous comparison.

### Survival and multivariate analysis for Truncated-O-glycan score

**Figure 2:**
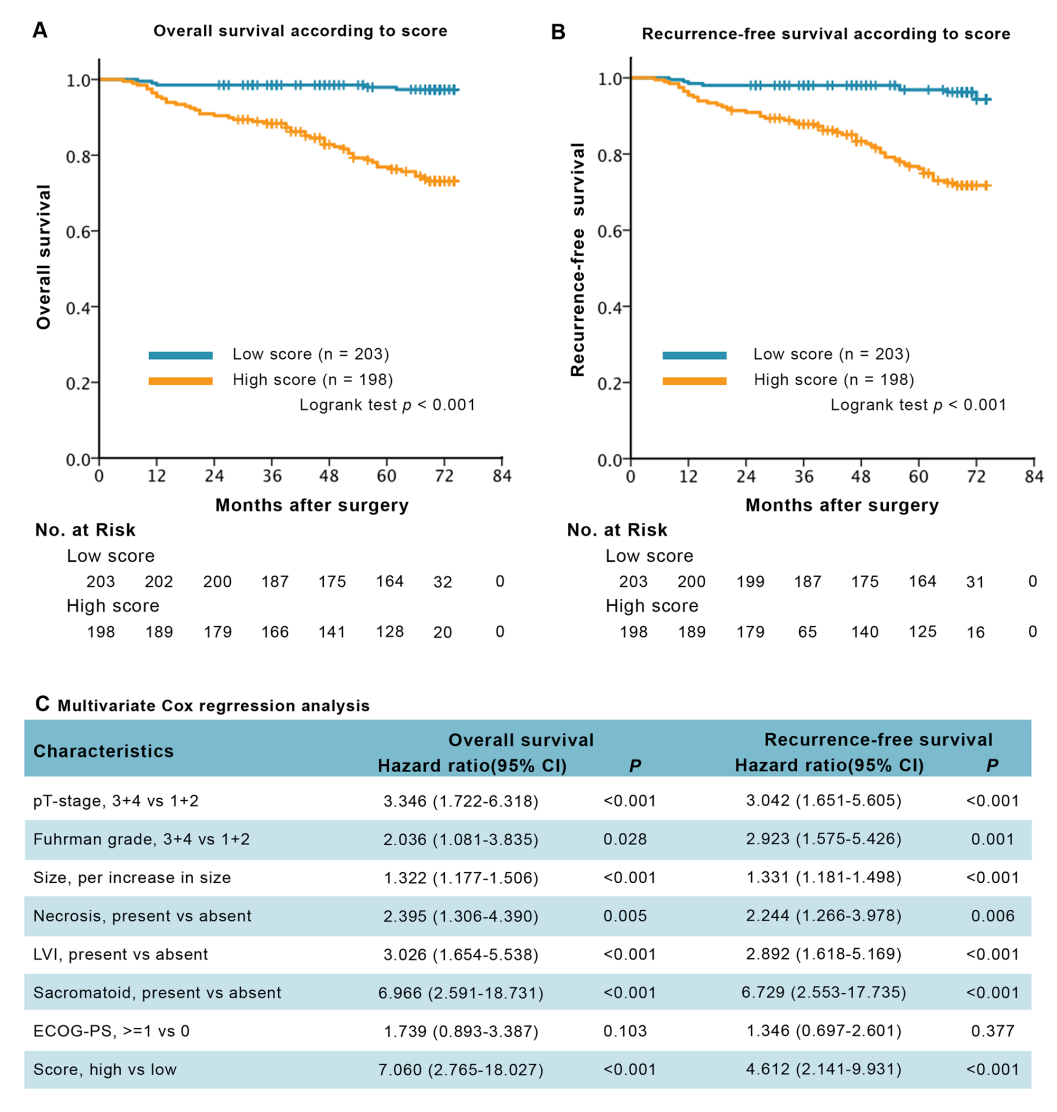
Survival analysis and multivariate analysis for overall survival (OS) and recurrence-free survival (RFS), according to Truncated-O-glycan score **A.**-**B.** Kaplan-Meier curves of OS and RFS according to the different level of Truncated-O-glycan score. **C.** Multivariate Cox regression analysis for OS and RFS. Note: gender and age at surgery were excluded from the multivariate analysis, *p* < 0.05 is considered statistically significant.

Patients with high Truncated-O-glycan score correlated with worse outcomes than those with low score (Figure 2A-2B), low rates of 5-year overall survival (76.3% *vs* 97.3%) and 5-year recurrence-free survival (76.3% *vs* 96.3%). The correlation remained significant in multivariate Cox regression analysis (Figure 2C) that included pT stage, Fuhrman grade, tumor size, necrosis status, lymphovascular invasive (LVI) status, sacromatoid status and ECOG-PS status. In the analysis of overall survival, the hazard ratio for death of Truncated-O-glycan score was 7.060 (95% CI: 2.765 to 18.027; *p* < 0.001); and in the analysis of recurrence-free survival, the hazard ratio for relapse was 4.812 (95% CI: 2.141 to 9.931; *p* < 0.001). The consistent result has been observed when evaluate Truncated O-glycans score as a continuous variable in the COX regression analysis in addition to as a dichotomous variable (low/high) (Supplementary Table S5).

**Figure 3:**
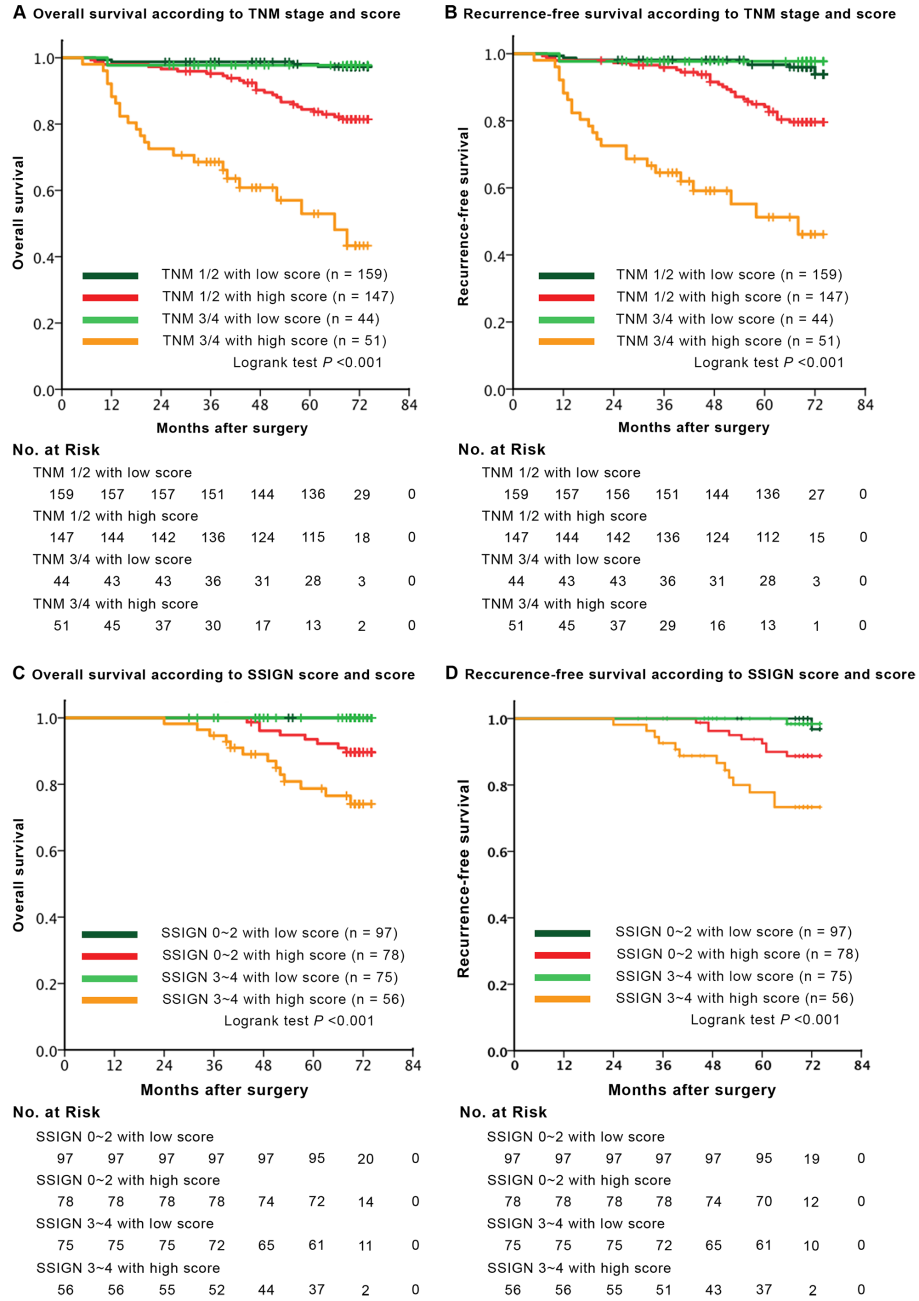
Survival analysis for overall survival (OS) and recurrence-free survival (RFS) according to Truncated-O-glycan score in each TNM stage and SSIGN category **A.**-**B.** Kaplan-Meier curves of OS and RFS according to the different level of Truncated-O-glycan score, subclassification with regard to TNM stage. **C.**-**D.** Kaplan-Meier analysis of OS and RFS according to the difference expression level of Truncated-O-glycan score, subclassification with regard to SSIGN score. Note: SSIGN score, Mayo Clinic Stage Size Grade and Necrosis score; *p* < 0.05 is considered statistically significant.

There are much interesting results when we further stratify overall cohort into subgroups according to Truncated-O-glycan score and TNM stage or SSIGN score (Figure 3). Patients with earlier TNM stages or lower SSIGN score associated high score displayed worse outcomes compared with those with low score associated advanced TNM stage or higher SSIGN score (*p* = 0.029). By conventional knowledge, patients in earlier TNM stage or lower SSIGN score general exhibit a better prognosis than those in advanced stage or higher score. However, there is no difference among such patient groups in low Truncated-O-glycan score group in our study (*p* = 0.987).

### ROC analysis for comparing the predicting sensitivity and specificity according to TNM stage and Truncated-O-glycan score

**Figure 4:**
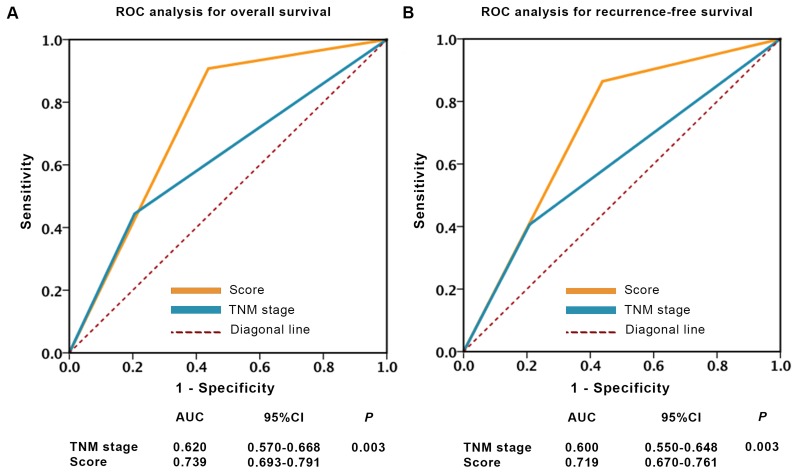
ROC analysis of clinical outcome prediction in patients with localized ccRCC **A.**-**B.** Sensitivity and specificity to predict overall survival and recurrence-free survival. Note: *p* values indicating statistical significance of ROC AUC of TNM stage *vs* Truncate-O-glycan, *p* < 0.05 is considered statistically significant.

As seen in Figure 4, the predicting sensitivity and specificity of Truncated-O-glycan score (AUC: 0.739, 95% CI: 0.693-0.791 for OS prediction and AUC: 0.719, 95% CI: 0.670-0.761 for RFS prediction, Figure 4) showed markedly higher accuracy than TNM stage (AUC: 0.620, 95% CI: 0.570-0.668 for OS prediction and AUC: 0.600, 95% CI: 0.550-0.648 for RFS prediction, Figure 4).

## DISCUSSION

To our knowledge, this study is the first report of the prognostic value of truncated-O-glycan expression to postoperative patients with localized ccRCC . Based on Tn-, sTn-, T- and sT-antigen prognostic value, we constructed a Truncated-O-glycan score for increased prognostic power. High score tumors were associated with worse prognosis than were low score tumors, with lower rates of 5-year overall survival risk and 5-year recurrence-free survival risk. The correlation remained significant in multivariate Cox regression of OS and RFS. Of particularly interest, there is no difference between low risk patients (TNM 1/2 stage or SSIGN 0-2) and high risk patients (TNM 3/4 stage or SSIGN 3-4) in low score group. High risk patients in low score group showed a better prognosis than those with low risk in high score group. Therefor, the Truncate-O-glycan score may help to identify more defined prognostic subgroups. Thus, we advocate for the utilize of Truncate-O-glycan score to better inform postoperative management. For instance, localized ccRCC patients with high score may need adjuvant therapy and a more frequent follow-up after surgery, even though they are low-risk patients according to TNM stage. Additionally, the predicting sensitivity and specificity of Truncate-O-glycan showed markedly higher accuracy than TNM stage. In our opinion, integrating Tn-score into the TNM staging system could significantly improve the predictive accuracy of TNM stage.

Over several decades, growing evidences indicated that aberrant expression of O-GalNAc glycan core structures was not observed in normal cells and tissues. Since terminal α-linked GalNAc on malignant cells was first described in 1960s, the mechanism related to Tn-antigen in malignancy transformation and progression has yet been clearly elucidated. As described above, the median H-score of sTn- and sT-antigen is much higher than Tn- and T-antigen, it can be hypothesized that Tn-antigen was mostly transformed into sTn- and sT-antigen which may have critical roles in localized ccRCC progression. As a result, the molecular mechanism behind the excessive expression of sTn- and sT-epitopes on ccRCC may correlates with the up regulation of Sialyltransferases. Otherwise, several studies had been observed the up-regulation of many types of Sialyltransferases on tumor tissue. Such studies had been also indicated a subgroup of I-type lectins, called Siglecs, specifically recognize many structural features of sialic acids, are found on innate immune cells. Siglec-15 preferentially recognizes the tumoral sTn-antigen and further suggests that Siglec-15 on macrophages may contribute to tumor progression by the TGF-beta-mediated modulation of intratumoral microenvironments [15].

Tn-antigen is characterized by present in mucins which commonly overexpress in several tumors. It can be hypothesized that, following tumor-expressed mucins combined to immunocyte (including macrophage, dendritic cells), Tn-antigen could be combined to macrophage galactose-type lectin (MGL) expressed by macrophage and dendritic cells [16-18]. Depending on whether additional dendritic-cell activation occurs, Tn binding may lead either to an immune response or immune tolerance [16-18]. Unfortunately, both immune tolerance or immune response were currently demonstrated as important regulators of tumorgenenesis, the latter was well established to possibly promote tumor angiogenesis and lymphoangiogenesis [19].

In recent decades, immunotherapy has become an outstanding treatment after surgery for patients with high risk ccRCC. The finding of these O-GalNAc glycan core structures in neoplasm has encouraged considerable effort toward the development of Truncated-O-glycan-based vaccine [16, 20]. The application of Truncate-O-glycan score is not only able to accurately differentiate prognostic subgroups more efficient, but also to find out a certain truncated O-glycan possibly plays a crucial role in a certain patient. A preselection of patients with Truncated-O-glycan score may contribute to the higher success rate of O-GalNAc-glycan-core-structure-based vaccine.

Although this is the first report about the association between Tn-, sTn-, T- and sT-antigen expression as well as Truncated-O-glycan score and clinical features in patients with localized ccRCC, however, our study is retrospective in nature and relatively small in the size of the study cohort. A multicenter, prospective study is needed to validate the accuracy of Truncated-O-glycan score as well as TNM stage in which Truncate-O-glycan was integrated in larger population in the future. Secondly, because of limited patients with advanced disease (nodes accumulation or metastasis), we are not able to fully assess the prognostic accuracy of Truncate-O-glycan in these patients. Finally, functional studies should be conducted to uncover the biological mechanisms of in Tn-, sTn-, sT-antigen in ccRCC.

In conclusion, we evaluated the truncated O-glycans in a cohort of 401 localized ccRCC patients. Higher levels of Tn-, sTn-, sT-antigen are associated with worsen outcomes. Tn-, sTn-, sT-antigen may contribute independent prognostic information. By combining the information of Tn-, sTn-, sT-antigen. Truncated-O-glycan score might become a promising biomarker for identifying prognostic subgroups with different risk precisely and maybe also guiding further therapy decisions.

## PATIENTS AND METHODS

### Patients database

All study protocols were permitted by The Clinical Research Medical Ethics Committee of Zhongshan Hospital of Fudan University (Shanghai, China) and were carried out in accordance with the approved guidelines. Written informed consent was obtained from each participant. Formalin-fixed paraffin-embedded (FFPE) tissue samples of 401 patients who diagnosed as localized ccRCC have been used in this retrospective study. Patient with localized ccRCC was defined as a patient with neither lymph nodes accumulation nor metastasis. All involved patients underwent partial nephrectomy or radical nephrectomy in Zhongshan Hospital between January 2008 and December 2009. The inclusion criteria for our study were as follows: (1) no history of anticancer therapy; (2) no history of other malignant neoplasm; (3) histopathologically proven ccRCC; (4) complete available follow-up data. We excluded the patients who had no FFPE tissue sample, without available follow-up data, lymph nodes invasive (N1), metastasis (M1), suffered from bilateral disease and familial RCC, received preoperative neoadjuvant and/or postoperative adjuvant therapy or died within the first month after surgery were excluded. Tumor stages were histologically classified according to 2010 AJCC TNM classification [21]. The follow-up was carried out postoperatively with physical examinations, laboratory studies, chest and abdominal imaging every 6 months for year and annually up to 5 years. Based on Tn, sTn, T, sT expression and Truncated-O-glycan level, all patients were grouped into low score and high score, respectively. Other features of each patient, including age, gender, tumor size, pT-stage, pN-stage, metastasis, Fuhrman grade, necrosis, lymphovascular invasion (LVI), sarcomatoid and Eastern Cooperative Oncology Group performance status (ECOG-PS), was obtained from record of overall cohort. Overall survival (OS) and recurrence-free survival (RFS) were calculated from the date of surgery to the date of death (or the last follow-up) or to the date of recurrence (or the last follow-up), respectively.

### Immunohistochemical testing

Bioinylated Jaclin (lectin for T-antigen, 1:15mg/ml, Vector Laboratories, U.S.A), Peanut Agglutin Jaclin (lectin for sT-antigen, 1:15mg/ml, Vector Laboratories, U.S.A), Vicia Villosa Lectin Jaclin (lectin for Tn-antigen, 1:15mg/ml, Vector laboratories, U.S.A) and anti-sialyl Tn monoclonal antibodies (1:30, Abcam, ab115957, Cambridge, MA) was applied for FFPE immunohistochemistry testing. The staining protocol was based on recommendation from Vector Laboratories (http://vectorlabs.com) and Nordic Immunohistochemical Quality Control organization (www.nordiqc.org). All of cases was stained by IHC-p at once. The staining score was evaluated by two independent pathologists without the knowledge of clinicopathological data. The expression intensity of Tn, sTn, T, sT was scored as 0 (negative), 1 (weak), 2 (moderate) and 3 (strong) and the staining extent was scored as the percentage of positive area in all intercellular space (0-100%). The staining intensity and extent were multiplied to generate an immunohistochemistry score (H-score) on a continuous scale of 0-300. We selected the optimum cutoff score for the expression of these antigens by using X-tile software version 3.6.1 (Yale University School of Medicine, New Haven, CT, USA) based on the association with the patients’ OS.

### Truncated-O-glycan score construction

Given that the prognostic power observed in any independent antigen is relative low, we therefor constructed a new weighted formula, named Truncated-O-glycan score. The weighted formula is the sum of H-score of Tn-, sTn- and sT-antigen and each H-score need to be weighted by a certain coefficient. Given that there is a strong correlation between sTn- and sT-antigen, we combined sTn- and sT-antigen before adding Tn-antigen to the weighted formula. This certain coefficient is the Cox regression coefficient of each antigen.

Further, we utilized X-tile plot analysis by apply X-tile to select the optimum cutoff score to separate patients into high and low Truncated-O-glycan score groups.

### Statistical analysis

X-tile plot analysis [22] was conducted to select the optimum cutoff of the H-score to stratified patients into low and high groups. The association between Tn-, sTn-, T-, sT-antigen expression, Truncated-O-glycan score and clinicopathological features were evaluated using Student’s t test, χ2-test, the Mann-Whitney test and Wilcoxon rank-sum test, as appropriate. The regression analysis was applied for validate the correlation between sTn and sT expression level. Survival analysis was established by Kaplan-Meier method by using Logrank test. Univariate and multivariate Cox regression analysis were applied to calculate the hazard ratios and 95% confidence intervals of clinicopathological features. Harrell Concordance index (C-index) was done to estimate the prognostic value between Tn, sTn, T, sT and Score. ROC analysis was constructed to compare the sensitivity and the specificity of predicting OS and RFS according to the variables. All statistical tests were two-tailed, and the differences were defined significant at level of *p* < 0.05. All statistical were accomplished by using X-tile software v3.6.1 (Yale University, New Haven, CT, USA), IBM SPSS Statistics 23.0 (IBM Corp, Armonk, New York), MedCalc Software 11.4.2.0 (MedCalc, Mariakerke, Belgium) and Graphpad Prism 6.0 (GraphPad Software, Inc).

## SUPPLEMENTARY MATERIALS FIGURES AND TABLES



## Abbreviations

RCC: Renal cell carcinoma
ccRCC: Clear cell renal cell carcinoma
IHC: Immunohistochemistry
FFPE: Formalin-fixed paraffin-embedded
LVI: lymphovascular invasion
OS: Overall survival
RFS: Recurrence free survival
ROC: Receiver operating characteristic
AUC: Area under curve
SSIGN: Mayo Clinic stage, size, grade and necrosis score
