# Supplementary material for: High truncated-O-glycan score predicts adverse clinical outcome in patients with localized clear-cell renal cell carcinoma after surgery
Source: Oncotarget. 2017 Mar 4;8(45):80083–92. doi: 10.18632/oncotarget.15900 (PMC5668123; doi:10.18632/oncotarget.15900)
Supplement: Supplementary file 1 [file oncotarget-08-80083-s001.pdf]

High truncated-O-glycan score predicts adverse clinical outcome in patients with localized clear-cell renal cell carcinoma after surgery

Supplementary Material

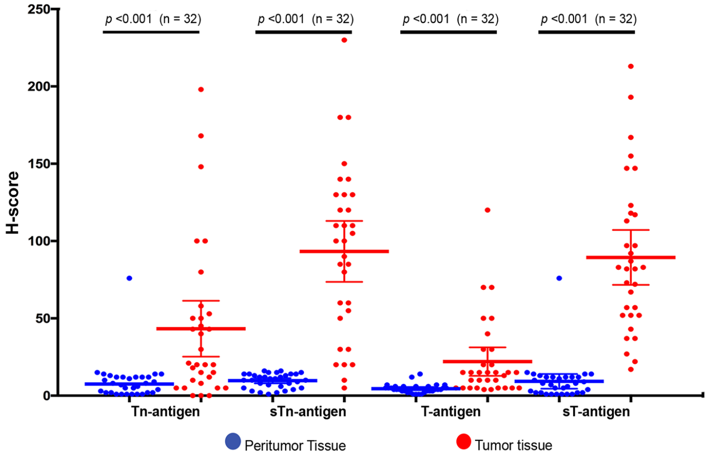

Figure S1. The expression of truncated O-glycans in tumors tissues and its corresponding peritumor tissues. Note: scatter plots for H-score in paired tumors (n = 32) and peritumors tissues (n = 32). Nonparametric Mann-Whitney test *p* value. *p* < 0.05 is considered statistically significant.

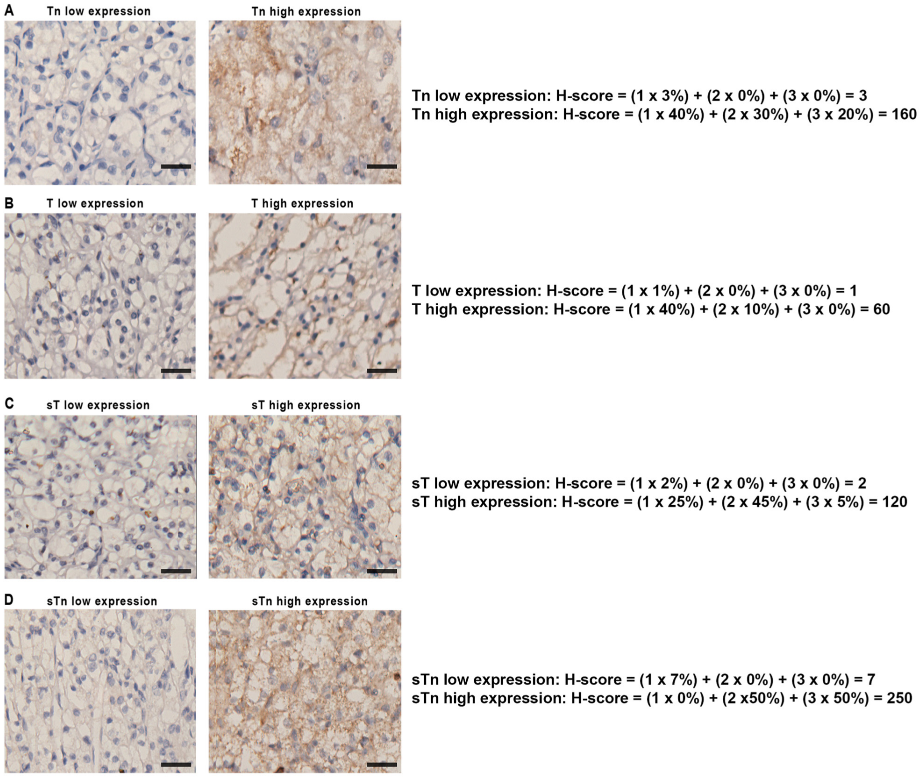

Figure S2. Representative immunohistochemical (IHC) images of ccRCC tissues with differential H-score of truncated O-glycans. Note: Scale bar: 40μm (original magnification x 200).

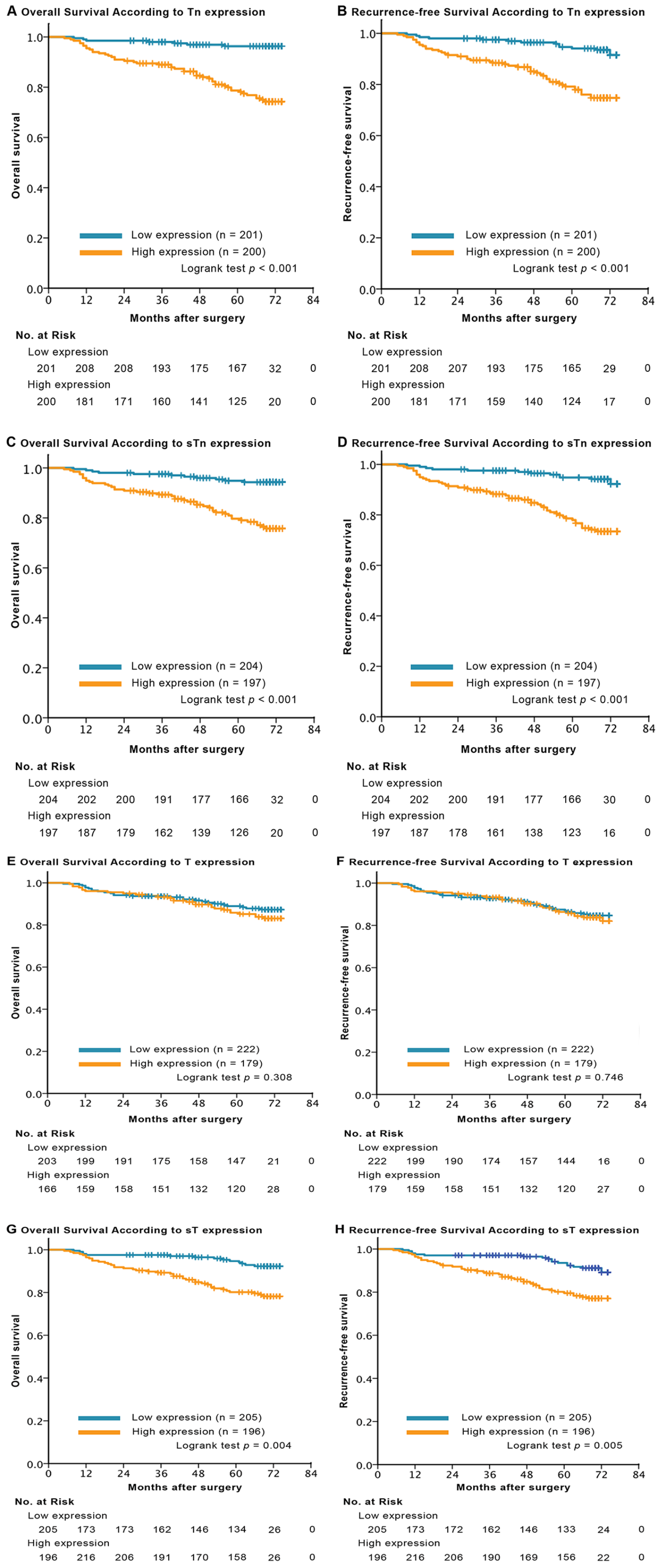

Figure S3. Survival analysis of overall survival (OS) and recurrence-free survival (RFS), according to the expression level of truncated O-glycans. Kaplan-Meier curves of OS and RFS according to Tn-antigen expression (A-B), sTn-antigen expression (C-D), T-antigen expression (E-F), sT-antigen expression (G-H). Note: $p < 0.05$ is considered statistically significant.

Table S1. Multivariate Cox regression model for overall survival and recurrence-free survival

Characteristics	Tn		sTn		sT	
	Hazard ratio (95% CI)	P^*	Hazard ratio (95% CI)	P^*	Hazard ratio (95% CI)	P^*
**Overall survival**						
Tumor size (per increase in size)	1.336 (1.182-1.510)	<0.001	1.346 (1.193-1.518)	<0.001	1.325 (1.170 ± 1.501)	<0.001
pT-stage (T3+T4 vs T1+T2)	3.872 (2.066-7.257)	<0.001	3.826 (2.077-7.047)	<0.001	4.000 (2.149 -7.445)	<0.001
Fuhrman grade (3+4 vs 1+2)	2.096 (1.102-3.988)	0.024	2.322 (1.225-4.402)	0.010	2.617 (1.375-4.980)	0.003
LVI§ (present vs absent)	2.775 (1.510-5.099)	0.001	3.155(1.722-5.780)	<0.001	2.720 (1.476-5.014)	0.001
Necrosis (present vs absent)	2.290 (1.243-4.219)	0.008	2.608 (1.430-4.757)	0.002	2.662 (1.451-4.885)	0.002
Sarcomatoid (present vs absent)	6.540 (2.430-17.602)	<0.001	7.759 (2.916-20.648)	<0.001	7.156 (2.639-19.402)	<0.001
ECOG-PS‡	1.597 (0.819-3.113)	0.169	1.319 (0.667-2.608)	0.425	1.707 (0.866-3.367)	0.123
Tn expression (high vs low)	4.016 (1.760-9.166)	0.001	-	-	-	-
sTn expression (high vs low)	-	-	3.008 (1.509-5.994)	0.002	-	-
sT expression (high vs low)	-	-	-	-	2.263 (1.208-4.239)	0.011
**Recurrence-free survival**						
Tumor size (per increase in size)	1.339 (1.190 – 1.506)	<0.001	1.336 (1.190-1.499)	<0.001	1.321 (1.172-1.489)	<0.001
pT-stage (T3+T4 vs T1+T2)	3.562 (1.950-6.508)	<0.001	3.351 (1.862-6.030)	<0.001	3.605 (1.978-6.570)	<0.001
Fuhrman grade (3+4 vs 1+2)	3.248 (1.728-6.105)	<0.001	3.211 (1.724-5.983)	0.010	2.552 (1.425-4.571)	0.003
LVI§ (present vs absent)	2.627 (1.471-4.692)	<0.001	3.054 (1.699-5.490)	<0.001	2.552 (1.425-4.571)	0.002
Necrosis (present vs absent)	2.259 (1.264-4.036)	0.006	2.467 (1.399-4.349)	0.002	2.470 (1.393-4.381)	0.002
Sarcomatoid (present vs absent)	6.715 (2.545-17-719)	<0.001	7.291 (2.780-19.125)	<0.001	6.841 (2.583-18.116)	<0.001
ECOG-PS‡	1.259 (0.650-2.438)	0.495	1.031 (0.526-2.021)	0.929	1.350 (0.691-2.363)	0.123
Tn expression (high vs low)	2.019 (1.049-3.885)	0.035	-	-	-	-
sTn expression (high vs low)	-	-	3.292 (1.697-6.385)	<0.001	-	-
sT expression (high vs low)	-	-	-	-	2.031 (1.137-3.629)	0.017

**P*-value <0.05 was regarded as statistically significant;
§LVI = lymphovascular invasion; ‡ ECOG-PS = Eastern Cooperative Oncology Group performance status.

Table S2. Multivariate Cox regression model for overall survival and recurrence-free survival

Factors	Hazard ratio (95% CI)	P*
**Overall survival**		
Tumor size (per increase in size)	1.318 (1.165 – 1.492)	<0.001
pT-stage (T3+T4 vs T1+T2)	3.596 (1.929 – 6.704)	<0.001
Fuhrman grade (3+4 vs 1+2)	2.047 (1.075 – 3.898)	0.029
LVI (present vs absent)	2.869 (1.545 – 5.327)	0.001
Necrosis (present vs absent)	2.370 (1.297 – 4.332)	0.005
Sarcomatoid (present vs absent)	6.339 (2.351 – 17.091)	<0.001
ECOG-PS‡	1.454 (0.731 – 2.890)	0.286
Tn-expression (high vs low)	3.054 (1.297 – 7.189)	0.01
sTn-expression (high vs low)	2.768 (1.357 – 5.645)	0.005
sT-expression (high vs low)	2.031 (1.137-3.629)	0.017
**Recurrence-free survival**		
Tumor size (per increase in size)	3.268 (1.804 – 5.920)	<0.001
pT-stage (T3+T4 vs T1+T2)	3.268 (1.804 – 5.920)	<0.001
Fuhrman grade (3+4 vs 1+2)	3.079 (1.636 – 5.796)	<0.001
LVI (present vs absent)	2.923 (1.606 – 5.319)	<0.001
Necrosis (present vs absent)	2.339 (1.321 – 4.140)	0.004
Sarcomatoid (present vs absent)	5.976 (2.227 – 16.033)	<0.001
ECOG-PS‡	1.086 (0.549 – 2.146)	0.813
Tn-expression (high vs low)	2.263 (1.208-4.239)	0.011
sTn-expression (high vs low)	3.246 (1.634 – 6.451)	0.001
sT-expression (high vs low)	2.755 (1.448 – 5.240)	0.002

Table S3. The correlation between score discontinuous and clinical characteristics.

Clinical Characteristics	Score		P^*
	Low score (n = 203)	High score (n = 198)	
Age at surgery, year			0.431
Mean ± SD[¶]	55.09 ± 12.43	56.96 ± 11.34	
Gender			0.056
Female	66	50	
Male	134	148	
Tumor size, cm			0.061
Mean ± SD[¶]	3.97 ± 2.10	4.65 ± 2.59	
pT-stage			0.234
pT1 + pT2	159	145	
pT3 + pT4	44	53	
TNM stage			0.336
Stage I + Stage II	159	147	
Stage III + Stage IV	44	51	
Fuhrman grade			**<0.001**
Grade 1 + grade 2	152	108	
Grade 3 +grade 4	51	90	
LVI[§]			0.080
Absent	161	143	
Present	41	55	
Necrosis			**0.010**
Absent	176	152	
Present	27	46	
Sarcomatoid			0.181
Absent	200	191	
Present	3	7	
ECOG-PS[‡]			0.349
0	175	164	
≥1	28	34	
Surgical type			0.935
Partial Nephrectomy	94	90	
Radical nephrectomy	104	102	
Laparascopic radical nephrectomy	5	6	

*P-value <0.05 was regarded as statistically significant;

[¶] The results of continuous variables are presented as mean ± SD (standard deviation);

[§]LVI = lymphovascular invasion; [‡] ECOG-PS = Eastern Cooperative Oncology Group performance status.

Table S4. Comparison of the predictive accuracy of the expression of Tn-, sTn-, sT-antigen and Truncated-O-glycan score.

**Model**	**Harrell concordance index**	
	**OS**	**RFS**
Tn H-score (continuous)	0.737	0.704
Tn (discontinuous)	0.696	0.661
sTn H-score (continuous)	0.705	0.732
sTn (discontinuous)	0.667	0.675
sT H-score (continuous)	0.686	0.676
sT (discontinuous)	0.638	0.629
Score (continuous)	0.752	0.741
Score (discontinuous)	0.720	0.706

Table S5. Multivariate Cox regression model for overall survival and recurrence-free survival (truncated O-glycans score modeled as continuous variable)

**Factors**	**Hazard ratio (95% CI)**	**P***
**Overall survival**		
Tumor size (per increase in size)	1.343 (1.191 – 1.515)	<0.001
pT-stage (T3+T4 vs T1+T2)	4.395 (2.340 – 8.254)	<0.001
Fuhrman grade (3+4 vs 1+2)	2.232 (1.160 – 4.295)	0.016
LVI (present vs absent)	2.591 (1.394 – 4.813)	0.003
Necrosis (present vs absent)	2.492 (1.343 – 4.625)	0.004
Sarcomatoid (present vs absent)	6.141 (2.237 – 16.857)	<0.001
ECOG-PS‡	1.432 (0.724 – 2.832)	0.302
Truncated O-glycans score (modeled as continuous variable)	1.678 (1.203 – 2.340)	0.002
**Recurrence-free survival**		
Tumor size (per increase in size)	1.335 (1.188 – 1.500)	<0.001
pT-stage (T3+T4 vs T1+T2)	3.888 (2.120 – 7.133)	<0.001
Fuhrman grade (3+4 vs 1+2)	3.206 (1.697 – 6.506)	<0.001
LVI (present vs absent)	2.441 (1.335 – 4.399)	0.003
Necrosis (present vs absent)	2.307 (1.287 – 4.134)	0.005
Sarcomatoid (present vs absent)	5.976 (2.227 – 16.033)	<0.001
ECOG-PS‡	1.151 (0.587 – 2.256)	0.682
Truncated O-glycans score (modeled as continuous variable)	1.593 (1.158 – 2.189)	0.004
